# “Every structure we're taught goes out the window”: General practitioners' experiences of providing help for patients with emotional concerns'

**DOI:** 10.1111/hsc.12860

**Published:** 2019-10-16

**Authors:** Daisy Parker, Richard Byng, Chris Dickens, Rose McCabe

**Affiliations:** ^1^ University of Exeter Exeter UK; ^2^ Plymouth University Plymouth UK; ^3^ City, University of London London UK

**Keywords:** Communication, Doctor‐patient relationship, Mental health, Patient‐centred care, Primary care, Qualitative analysis

## Abstract

Up to 40% of general practitioners (GP) consultations contain an emotional component. General practitioners (GPs) have to provide care with limited time and resources. This qualitative study aimed to explore how GPs care for patients experiencing emotional concerns within the constraints of busy clinical practice. Seven GPs participated in three focus groups. Groups were recorded, transcribed and analysed thematically. Three themes were identified. (a) Collaboratively negotiated diagnosis: How patients' emotional concerns are understood and managed is the result of a negotiation between patient and GP belief models and the availability of treatments including talking therapy. (b) Doctor as drug: Not only is a continuous relationship between GPs and patients therapeutic in its own right, it is also necessary to effectively diagnose and engage patients in treatment as patients may experience stigma regarding emotional concerns. (c) Personal responsibility and institutional pressure: GPs feel personally responsible for supporting patients through their care journey, however, they face barriers due to lack of time and pressure from guidelines. GPs are forced to prioritise high‐risk patients and experience an emotional toll. In conclusion, guidelines focus on diagnosis and a stepped‐care model, however, this assumes diagnosis is relatively straightforward. GPs and patients have different models of psychological distress. This and the experience of stigma mean that establishing rapport is an important step before the GP and patient negotiate openly and develop a shared understanding of the problem. This takes time and emotional resources to do well. Longer consultations, continuity of care and formal supervision for GPs could enable them to better support patients.

**What is known about this topic**

- Previous research has highlighted the role of general practitioners (GPs) in detecting and managing emotional concerns and the therapeutic role of the doctor–patient relationship.
- GPs face a number of barriers to providing guideline‐concordant care in practice.

**What this paper adds**

- Establishing a therapeutic relationship supports GPs and patients to negotiate openly and develop a shared understanding of the problem.
- NICE guidelines relating to emotional concerns should acknowledge the importance of a flexible approach to diagnosis and treatment.
- Longer consultations, continuity of care and formal supervision for GPs would help them to provide better care for patients experiencing emotional concerns.

## 1 INTRODUCTION

Mental health problems are one of the main causes of disease burden worldwide (Vos et al., 2015), and it is estimated that mental health problems cost the UK economy up to £100bn a year (Davis, 2014). With 40% of primary care consultations having a psychosocial component (Mind, 2018), general practitioners (GPs) are the most frequently used providers of mental healthcare in the UK. The mental health problems faced in primary care are heterogeneous, undifferentiated and present as a continuum with symptoms of different diagnoses often inextricably linked (Cape, Barker, Buszewicz, & Pistrang, 2000; Gask, Klinkman, Fortes, & Dowrick, 2008). Due to this complexity, this study uses the term ‘emotional concerns’ throughout to reflect the patients most commonly seen by GPs.

GPs report feeling responsible for, and engaged in, the identification and management of patients experiencing emotional concerns (Liu, Lu, & Lee, 2008). However, GPs working in the UK have to manage a number of pressures which make these consultations more challenging. Short consultations, feelings of low self‐worth, and stigma, can deter patients from presenting emotional concerns to their GP (Dew, Dowell, McLeod, Collings, & Bushnell, 2005; Gask, Rogers, Oliver, May, & Roland, 2003; Kadam, Croft, McLeod, & Hutchinson, 2001; Wheat, Barnes, & Byng, 2015). Difficulties communicating with secondary care services can make GPs feel unable to direct patients to appropriate support (Cohen, 2008).

Current guidance for the identification and management of emotional concerns in primary care, specifically NICE guidance and the Quality and Outcomes Framework (QOF), prioritises the use of screening questions and a stepped‐care approach (National Institute for Health and Care Excellence (NICE), 2009, 2011). However, despite these guidelines, rates of antidepressant prescribing have doubled in the last 10 years (Bullard, 2017), and GPs report that the QOF is a ‘box‐ticking’ exercise that draws them away from patient‐led consultations (Maisey et al., 2008).

Previous research has found that GPs often consider patients’ emotional concerns to be related to life stress (Dew et al., 2005; Johnson, Williams, Macgillivray, Dougall, & Maxwell, 2017; Thomas‐MacLean, Stoppard, Miedema, & Tatemichi, 2005), and that instead of using diagnostic tools, GPs report relying on intuition and rapport building. This suggests that GPs may understand and manage emotional concerns in primary care using practices that are not recognised in current guidelines (National Institute for Health and Care Excellence (NICE), 2009, 2011).

Therefore, it is important to develop a better understanding of how GPs are managing these consultations in practice. This study aimed to explore GPs’ experiences of providing care for patients experiencing emotional concerns, focusing on the research questions: (a) what are GPs’ experiences of providing care for patients with emotional concerns? (b) what approaches do GPs use that may differ from the guidance?, and (c) how do GPs provide care within the constraints of busy clinical practice?

## 2 METHODS

### 2.1 Terminology

In GP consultations, mental health problems may be understood by GPs and patients in various ways and also encompass a broader range of problems than diagnosed mental health disorders. Hence, in this study, the term ‘emotional concerns’ is used to represent this diversity of experiences and understandings across patients and practitioners and includes; (a) common mental health problems, specifically anxiety and depression, (b) undifferentiated low mood, stress and/or anxiety that may be subclinical or not formally diagnosed, (c) low mood, stress and anxiety that may be attributed to difficult life circumstances.

### 2.2 Design

The study is part of a wider project that aims to develop an intervention to support GPs when communicating with patients with emotional concerns. Focus groups were used to facilitate the unearthing of topics that were not previously considered by the researchers. Compared to individual interviews, focus groups have a more naturalistic interaction and group dynamics can facilitate disclosure (Barbour, 2007; Farquhar & Das, 1999; Wilkinson, 2004). Focus groups allow participants to build on each other’s contributions or challenge each other’s statements, leading to the production of more elaborate accounts than would be gained by doing individual interviews (Steward & Shamdasani, 2015).

### 2.3 Recruitment

An email introducing and describing the study was sent to nine practicing GPs in Devon and the East Midlands. The email explained that the study would involve attending one focus group to explore GP experiences of providing help for patients experiencing emotional concerns. GPs who were interested in taking part in the study were asked to invite colleagues from their surgery to a focus group. GPs were targeted to achieve a variation in demographic characteristics, specifically variation in location of their practice, gender and age. Ethical approval was granted by the University of Exeter Medical School Research Ethics Committee (Reference: 16/11/111) prior to the commencement of the study.

### 2.4 Procedure

Focus groups were conducted between March and August 2017. Participants were given a detailed information sheet about the study before giving consent. Written informed consent was provided by all participants before the start of each focus group. All of the focus groups took part in the participants’ surgeries at a time that suited them. Participants took part in one focus group with one or two other GPs from the same practice. Three focus groups were conducted in total. The groups were facilitated by DP and a second researcher acted as co‐facilitator. All focus groups were audio‐recorded using two digital voice recorders. Participants were informed of their right to withdraw from the study at any time. Due to the potentially distressing nature of the topic, a standardised risk assessment protocol was in place should participants become distressed. The risk assessment protocol was to be used if any participant disclosed thoughts of self‐harm and included standardised questions and a flowchart of actions questions to assess and manage risk of self‐harm. Fortunately, no participants became distressed during, or as a result of, the focus groups.

### 2.5 Topic guide

The discussion followed a semi‐structured topic guide which was designed to elicit areas of interest while also allowing participants to expand on their narratives and topicalise areas of personal importance. Questions were designed to allow participants to give a free narrative and build on one another’s responses. The topic guide was developed around three key areas: (a) GPs’ experiences of providing help for patients experiencing emotional concerns, (b) what techniques GPs use that may differ from the NICE guidance, and (c) how they provided care within the constraints of busy clinical practice. The topic guide was iteratively developed based on evidence from previous studies about mental health in primary care, and clinical and research experience of the research team. The topic guide was refined and revised after each focus group to consider topics introduced as important by participants.

### 2.6 Data analysis

Focus groups were transcribed verbatim and anonymised. Transcripts were analysed using inductive thematic analysis in accordance with guidelines recommended by Braun and Clarke (Braun & Clarke, 2006; Miles & Huberman, 1994). Transcripts were organised and managed using qualitative data analysis software NVivo 11 (QSR International, 2012). All transcripts were initially analysed independently by DP. First, familiarisation with the data was achieved by transcribing and checking the transcripts. Secondly, all of the transcripts were coded line‐by‐line. These codes were organised into categories which were considered in the context of the wider transcripts. Researchers considered what topics and processes clustered together and which were distinctly different. Categories were iteratively refined using a constant comparative process, moving from descriptive categories to conceptual themes and subthemes. Maps and diagrams were used throughout to interrogate the relationships between themes. The developing analysis was discussed with RM and RB throughout to develop consensus about the analysis and ensure reliability of the analysis. Data were also presented at regular qualitative data sessions.

## 3 FINDINGS

Three of the nine GPs approached responded to the email about the study. These three GPs invited between one to two colleagues each from their practice to participate in a focus group. Three focus groups were conducted lasting on average 49 minutes. Seven GPs participated in total. GPs were from practices based in the East Midlands and the South West of England. Three GPs were based in rural practices, two from semi‐rural, and two were from urban practices. Two were male and five were female. GPs ranged from newly qualified to over twenty‐five years in practice. Participant and focus group details can be found in Tables 1 and 2 respectively.

**Table 1:** Profile of participants

Characteristics	No. of participants
Sex	
Male	2
Female	5
Location of practice	
Rural	3
Semi‐rural	2
Urban	2

**Table 2:** Focus group characteristics

	Participants	Female participants	Male participants	Length (minutes)
Focus group 1	3	2	1	62.42
Focus group 2	2	2	0	42.47
Focus group 3	2	1	1	41.05
Total	7	5	2	145.31

Three themes were identified. (a) Collaboratively negotiated diagnosis. How patients’ emotional concerns were understood and managed was the result of a negotiation between patient and GP belief models and the availability of treatments. (b) Doctor as drug: Not only was a continuous relationship between GPs and patients therapeutic in its own right, but it was also necessary to effectively diagnose and engage patients in treatment. (c) Personal responsibility and institutional pressures: GPs took personal responsibility for providing effective care for patients, however lack of time and pressure from guidelines forced them to prioritise higher risk patients. Number of references for each theme can be found in Table 3.

**Table 3:** Number of references for themes and subthemes

Collaboratively negotiated diagnosis	233
Patient’s expectations, understandings and preferences	97
GP’s beliefs, style and preferences	67
GP and patient	21
Treatment availability	42
Doctor as drug	105
Different role for psychological consultations	24
Continuity	28
Relationship supports consultation	11
Stigma makes help seeking and disclosure difficult	31
Personal responsibility and institutional pressure	158
Constraints of guidelines	20
Risk assessment	47
Emotionally draining	55
Time pressure – mental healthcare takes time to do well	32

### 3.1 Collaboratively negotiated diagnosis

How GPs understood and managed their patients’ emotional concerns was not a straightforward process and did not rely on diagnostic tools and ‘textbook’ symptoms. Instead, emotional concerns were understood as the result of an interaction between patient factors, GP factors, and availability of treatment. Firstly, patient’s preferences, expectations, understandings, and social context were all important for guiding diagnostic and treatment decisions.

You get [patients] who do want a diagnosis, and they want it to be a problem, a condition. If that’s how they deal a bit better, then I go into detail about pathophysiology and the underlying chemical changes. I guess it works well for those people. (GP5, female)

As there is no blood test to diagnose emotional concerns, GPs needed to be skilled in eliciting accurate information from patients. However, GPs reported that it was often challenging to uncover the ‘true’ problem. This was often the case when patients did not understand their experiences. In these cases, GPs reported using techniques such as allowing for silence, normalising and using visual aids to develop a joint understanding of the problem.

You can try phrasing questions in a different way to work around that block… active listening to show that you’re taking what they’re saying sensitively and seriously, and then they might then feel that they can tell you because you’re going to listen to what they’re saying. (GP6, male)

GPs felt that some patients may be deliberately deceptive, with GPs reporting that a small minority of patients may overstate the severity of their emotional concerns to increase their access to support. GPs reported the importance of experience and clinical intuition when determining the severity of patients’ distress.

It’s quite nuanced because you have to know that they’re manipulating you, and they’re a minority, but as an experienced GP you learn when to actively ignore certain little hidden agendas. (GP1, female)

GPs’ beliefs, style, and preferences affected how they understood their patients’ emotional concerns. This in turn affected the approach that they took when supporting these patients. Some GPs preferred a more involved approach, with one GP gaining a diploma in cognitive behavioural therapy. Other GPs, however, felt that patients’ emotional concerns were socially caused and therefore less appropriate for general practice. These GPs were more likely to take a signposting and referral approach.

I think that some GPs might see someone start talking about something psychological or to do with their wellbeing or their stress levels, and immediately go into mental health and diagnosis and treatment mode. Other styles might be more supportive coaching or “let’s give it a few appointments and see if there’s really a mental health issue or if this is just life stress”. (GP4, female)

GPs differed in whether they perceived emotional concerns to be a medical problem. Many GPs were concerned about medicalising ‘normal life stress’.

Their problems are mainly social, not medical, so we have to be aware of them because they’re causing social problems. (GP6, male)

As a result, GPs stressed the importance of understanding patients from a holistic perspective. Emotional concerns cannot be separated from the patients, so it was important to understand and explore patients’ symptoms and social circumstances. GPs’ awareness of their patients’ social circumstances, and the effect on their psychological well‐being, meant that GPs in this study used social prescribing.

…Social prescribing, where you’re sort of saying “get out there”, and whether it’s just walking your dog or getting some exercise at a class. (GP1, female)

Some GPs in this study reported basing their diagnostic and treatment decisions around patients’ preferences. Other GPs adopted a more directive approach. GPs reported that patients resisted antidepressants due to fears about becoming addicted. These GPs stated that if they had a ‘strong suspicion’ that a patient would benefit from taking antidepressants then they would be more directive and attempt to persuade the patient. While GPs recognised that it was ultimately the patient’s choice if they took the medication, they would ‘strongly encourage’ patients to try antidepressants.

[Patients] don’t take [antidepressants] because they mistrust them, other times they’re just in denial that there’s anything wrong… Sometimes if they don’t want to take any medication you say “well how about you just give it a trial because you’re going to know in two, three, four weeks whether it’s going to be effective” and then at three weeks you see them again and usually they’ve turned a corner. (GP1, female)

Finally, patients’ emotional concerns were sometimes understood and managed based on what treatment was available to them. GPs sometimes prescribed antidepressants because the waiting list for talking therapy was long and patients found it challenging to access. While the guidelines do not recommend antidepressants for mild depression, GPs sometimes felt that prescribing antidepressants was all they could do, and therefore judged that the potential benefits of antidepressants outweighed the risk of side effects.

Counselling has got quite a long waiting list, with people with low mood and depression and anxiety they’ve probably spent a few months contemplating coming, they’ve got up the courage to come, and then saying “oh yeah you can see a counsellor in three months” isn’t what they were hoping for, which can then lead to their mood going even further down. So then you think, actually they’d like antidepressants, they feel they’ll help, yeah there’s side effects, but on balance it’ll probably do them more good than harm. But it’s not the guideline. (GP7, female)

### 3.2 Doctor as drug

GPs emphasised the GP–patient relationship in consultations with patients experiencing emotional concerns. GPs highlighted the importance of building rapport, being supportive and providing holistic, person‐centred care.

That first consultation is often terribly therapeutic, they’ve let it all out you’ve shown some sympathy and sometimes you don’t need to do much more the second time. (GP1, female)

This was in contrast to the role GPs took with patients with acute medical concerns, which was more disease‐focused. The role of the GP–patient relationship in these consultations was considered to be an essential, core component. It was the contact with the GP themselves, as opposed to what a GP could do clinically (i.e. provide a referral or antidepressants), that formed the basis of treatment itself.

That’s probably the mode you go into where you think these eight minutes might make a difference… whereas if it’s a physical illness there are more facts involved and let’s do a test let’s find a right diagnosis let’s give you the right tablets. (GP4, female)

In addition to forming the basis of treatment itself, a GP–patient relationship was also useful clinically; having a good relationship with patients can support processes in the consultation such as identifying distress and persuading a patient of a diagnosis and/or to take medication. This relationship could also attenuate the effects of stigma which can make it difficult for patients to open up. GPs reported that patients will often present with a physical concern which is less stigmatised, and only disclose their emotional agenda after they have built up rapport and trust with their GP. An existing GP–patient relationship meant that the patient would already trust the GP and thus feel more able to disclose emotional concerns outright.

It does help when you know the patient very well, because if you’ve known them for donkey’s years they can come through the door and you can see that they’re depressed because you know them like you know a friend almost… it’s much easier then, whereas if you haven’t seen somebody before that’s more challenging. (GP1, female)

GPs may be the only healthcare professional that patients have a long‐term relationship with. GPs discussed concerns about referring patients to other healthcare professionals with whom the patient may not have the same rapport.

There is an opportunistic window when [the patient has] opened up to you, you’ve got that rapport, and there’s a possibility that you could refer them to somebody else with whom they don’t have that rapport, and then the chance to help them has gone. I always try to hold on to them a little bit, until I know that they’re in a safe pair of hands and they feel comfortable. (GP5, female)

All of the GPs in this study discussed the therapeutic relationship as central to the care that they provided patients and they attempted to maintain this relationship with patients. However, as discussed below, this was often difficult to achieve.

### 3.3 Personal responsibility and institutional pressure

GPs felt a personal responsibility to provide high‐quality care for patients. All GPs emphasised how much they cared for their patients and this resulted in GPs consistently going ‘above and beyond’. For example, GPs would set reminders to check that patients had attended follow‐up appointments, or ask patients who needed more time to come back at the end of the day. GPs would take personal responsibility for following the patient through their care.

I think if you’re the GP that they’ve come to see and you can see there’s a situation and you’re worried about it you just keep them coming back to see you until you can see that they’re out of the woods… And if you’re worried you put on a little reminder to check that they’ve been back. (GP1, female)

However, going above and beyond placed additional pressure on GPs. GPs experienced tensions between providing the care that they considered necessary, and time restrictions. Consultations with patients experiencing emotional concerns took time to do well, and these consultations often overran.

You have to overrun and it takes as long as it takes, especially if they’re really suicidal you take as long as it takes, but you can see your screen filling up and the numbers going up, five, six, seven patients waiting. (GP1, female)

While GPs stressed the importance of ‘taking as long as it takes’, GPs also discussed their techniques for managing time pressure. First, a pre‐existing relationship with the patient can expedite the consultation. Second, GPs keep the consultation shorter if they determine that the patient is at a low risk of self‐harm. Patients who the GP believes are at a more immediate risk of self‐harm need to be ‘talked down’, which takes longer.

To do it properly it does take longer, but you’ve got to try and tick those boxes. Prior knowledge of the patient helps a bit, you can cut corners a little bit if you know the patient. (GP6, male)

Time pressure in consultations meant GPs had to decide what to prioritise in these consultations. Often this was risk assessment. GPs discussed how important it is to not miss something serious, such as suicidal ideation or psychosis, meaning that risk assessment often takes priority in consultations over therapeutic work.

The first thing you’ve got to do is to suss out how severe their condition is, is it something to immediately worry about? Once I’ve sorted that out in my mind, then I can take a step back and work out what to do next. (GP1, female)

GPs were trained to ask standardised questions exploring suicidal ideation and were comfortable asking patients these questions directly. However, some GPs had received no formal mental health training beyond this. If GPs had training, it was mostly from placements on psychiatric wards, where training focused on risk assessment instead of providing therapeutic care. The patients in these wards were very different to patients seen in primary care.

[On psychiatric wards] you had to risk assess to the point of making a decision about admission and discharge, whereas we don’t get that level of stuff in general practice. (GP4, female)

Another competing pressure for GPs’ time was from the Quality and Outcomes Framework (QOF) to use the Patient Health Questionnaire (PHQ‐9). GPs in this study did not use these standardised measures as a diagnostic tool. For patients with emotional concerns, consultations often deviated from the standard consultation structure, meaning that guidelines become less useful.

We were supposed to be using [the PHQ‐9] for all patients… partly attached to the QOF (Quality and Outcome Framework). We’ve moved back from not using it with patients now because it can actually disrupt the conversation that you’re having. (GP2, female)

The tension between personal responsibility to support a patient, and institutional pressures, meant that consultations with an emotional agenda could be emotionally draining. GPs in this study received no formal support but did utilise a number of personal strategies. These included talking with family and colleagues, exercise and writing a diary.

I think you just have to be aware that if you have two or three big emotional consultations in a day‐ because a lot of your consultations you won’t find them emotionally draining if they’re about athlete’s foot or something ‐but if you do and you’re told a lot, or you end up providing a lot of support, and I have learned that I need to know that that’s the sort of day I’ve had. (GP4, female)

## 4 DISCUSSION

GPs and patients have different models of emotional concerns, meaning that how patients’ concerns are understood and managed in the consultation is the result of more than just symptom count and severity. The stigma associated with emotional concerns means that interpersonal rapport is important. This rapport not only supports the consultation but is also intrinsically therapeutic. Finally, consultations take time and emotional resources to do well. GPs take a personal responsibility for providing high‐quality care, however they often have to prioritise tasks such as risk assessment.

### 4.1 Strengths and limitations

This study must be considered in the context of its limitations. As with all participant report methods, there is a risk of recall bias when using focus groups. Observation of consultations, or tape assisted recall, may reduce the risk of recall bias.

Participants were from urban, semi‐rural and rural practices, had a wide range of clinical experience, and males and females were represented. However, participants were self‐selecting and therefore may have been more likely to be interested and engaged in mental healthcare and hold particularly strong views.

It was challenging to recruit busy GPs. The sample is small and may not be representative of the wider GP population. However, practices were targeted in order to get a spread of urban, rural and semi‐rural practices which included a broad range of experiences. As the sample was small, it was difficult to recruit an ethnically diverse sample. A broader recruitment area and using maximum variation sampling may have reduced this limitation and should be considered for future research.

### 4.2 Comparison with existing literature and implications for practice

These findings have implications for understanding how GPs help patients experiencing emotional concerns, in particular highlighting how current guidance is (and is not) utilised in general practice. Participants’ reports of ‘what works well’ in these consultations are summarised in Table 4.

**Table 4:** Participant reports of ‘what works well’

What works well	Strategies	Quotes
Build rapport	Allow patient to 'let it all out' Be sympathetic Active listening Avoid attending to computer	“That first consultation is often terribly therapeutic isn’t it, because it’s taken them an awful lot to come to the doctor in the first place and then they’ve let it all out you’ve shown some sympathy and sometimes you don’t need to do much more the second time” (GP1)
Elicit patient expectations	Elicit treatment preferences and expectations early Attend to patient's cues and clues Asking direct questions	"I try to engage what the patient wants early on, like some of them say "what’s wrong with me I think I’ve got depression I’ve done loads of reading", and if that’s the model they want to use you can talk about that." (GP5)
Reassure and validate	Reassure patients that they are not 'being silly' Validate emotional distress as a valid reason for seeking help from the GP	"I always say that we do have emergency appointments if you are in a crisis, and I just emphasise that it’s not for physical health it’s also for mental health… and I think it’s important that they know actually mental health is just as important" (GP7)
Help patient to help themselves	Give patients self‐help resources Use online resources such as Mood Gym Psychoeducation Social prescribing	"I think it would be very helpful because it just gives them something to do in the meantime and I think it probably would in a way reduce them the amount of consultations we have with them in the long run probably." (GP6)
Optimise continuity	Personally book patient’s follow‐up appointment See patient until s/he starts talking therapy	"I always try to hold on to them a little bit until I know that they’re in a safe pair of hands which they feel comfortable" (GP5)

The findings from this study reflect, and build on, existing literature. GPs experience tensions between what they believe is high‐quality care, and what they are able to achieve in practice. Firstly, many GPs in this study reported understanding patients’ emotional concerns as due to life stress. This is reflected in the literature, where GPs report tensions between being trained to approach emotional concerns as a biomedical issue, and their own beliefs that emotional concerns result from life stressors (Dew et al., 2005; Johnson et al., 2017; Thomas‐MacLean et al., 2005). As a result, some GPs preferred to utilise a multifaceted treatment approach including social prescribing (Johnson et al., 2017).

However, as rates of antidepressant prescribing have doubled in the last 10 years (Bullard, 2000), this study suggests that there is a disconnect between how GPs understand emotional concerns and how they treat them. One explanation for this may be limited access to other treatment options. GPs in previous studies have discussed feeling unable to refer patients to secondary services due to unacceptably long waiting lists, rigid criteria and high thresholds (Dew, Fox, Rodham, Taylor, & Harris, 2016; Saini, Chantler, & Kapur, 2015; Saini et al., 2010). Limited treatment options led to GPs being more likely to prescribe antidepressants, as this is the treatment over which they have the most control and they often felt that there was little else they could offer (Hyde et al., 2005; Saini et al., 2015; Saini, Chantler, & Kapur, 2018). As highlighted by Saini and colleagues (2015), this is seen as unacceptable to both GPs and patients, as the lack of choice infringes on the patients’ right to make decisions about their medical care. As a result, the choice that is often made by GPs and patients is between medication and no treatment at all. This is in sharp contrast to the NICE guidelines for depression and anxiety which recommend a stepped‐care approach (National Institute for Health and Care Excellence (NICE), 2009, 2011).

The contrast between what is recommended by the guidelines and what can be delivered in practice speaks to the need for guidelines to be evidence‐based and have an understanding of the pressures that GPs are under. GPs and patients need to be able to choose the treatment strategy that aligns with their understanding of the concern, and therefore improved access to psychological therapies and greater provision of social prescribing is important if the guidelines are to be followed.

The second tension that GPs face is developing and maintaining rapport with patients, while also achieving necessary institutional tasks with limited time. The importance of the GP–patient relationship is well understood in previous literature (Cape et al., 2000; Dew et al., 2016; Johnston et al., 2007; Strachan et al., 2015). More than half of GPs believe that the consultation, even without medication or a referral, is treatment in its own right (Dew et al., 2016; Strachan et al., 2015) and this is supported by evidence demonstrating an association between patients’ relationship with their GP and a reduction in symptom severity three months later (Cape, 2000). There are many ways GPs can build a relationship with their patients, including expressing warmth, empathy, respect and concern (Dowrick, 2000; Shattell & Starr, 2007).

However, GPs reported that time pressures reduced their ability to develop and maintain a therapeutic relationship. The potential for a consultation about emotional concerns to last longer puts pressure on both GPs and patients (Dew et al., 2016; Strachan et al., 2015). Short consultations mean that GPs have to prioritise risk assessment over therapeutic work. GPs were concerned about the possibility of missing something serious, such as suicide risk or psychosis (Thomas‐MacLean et al., 2005).

This has implications for future research. There is some evidence that suggests it is possible for GPs to develop and maintain a therapeutic relationship despite time pressures. When GPs pick up on patient cues and provide empathic responses, consultations are shorter (Levinson, Gorawara‐Bhat, & Lamb, 2000). Additionally, if GPs appear unrushed and convey empathy, even short consultations are therapeutic (Pollock & Grime, 2002). Therefore, it may be possible to support GPs to use their limited time in this way, even though both GPs and patients would benefit from longer consultations. Future research should explore other ways for GPs to manage these consultations with limited time.

Consultations with patients with emotional concerns were potentially draining. However, GPs reported not receiving formal support. Burnout, time pressure and feeling unable to provide patient‐centred care are key reasons why GPs decide to leave general practice (Doran, Fox, Rodham, Taylor, & Harris, 2015). GPs who feel pessimistic about their ability to support patients, or find managing these patients stressful and unrewarding, are less willing to be actively involved in supporting them (Ross, Moffat, McConnachie, Gordon, & Wilson, 1999), and more likely to identify barriers to treatment (Dowrick, Gask, Perry, Dixon, & Usherwood, 1999; Richards, Ryan, McCabe, Groom, & Hickie, 2004). Being overworked can reduce GPs’ ability to be compassionate and can increase feelings of anxiety and low mood (Riley et al., 2018). GPs in this study did not receive formal support, but highlighted their use of self‐care and informal support from colleagues. This has been highlighted as important by GPs in other studies (Pavlič, Treven, Maksuti, Švab, & Grad, 2018; Saini, Chantler, While, & Kapur, 2016). Accessing formal support for difficult experiences is challenging for GPs due to the lack of provision. This study adds to the evidence that GPs require support similar to that which is provided to mental health professionals.

However, merely supporting GPs to deal with the emotional toll of these consultations is insufficient. These focus groups highlight that GPs are passionate about providing patients with quality care, however, are constrained by time and guidelines that do not value the central role of contact with a GP. This study highlights the need for GPs to be able to work flexibly to provide the care that they feel is important.

## 5 CONCLUSION

This study has highlighted an incongruence between the care that GPs want to provide, what the guidelines suggest, and what is possible given short consultations and limited treatment options. GPs have developed a number of strategies to provide high‐quality care for patients given the constraints they are under. Future work should assess the utility of current guidelines and investigate ways of supporting GPs to provide high‐quality care within the primary care context.

## CONFLICT OF INTEREST

There are no conflict of interest to report.

## Funding information

The Judi Meadows Memorial Fund, a protected fund of the McPin Foundation, and the University of Exeter College of Medicine and Health jointly funded this study.

## ETHICAL APPROVAL

This project was reviewed and approved by the University of Exeter Medical School Research Ethics Committee. UEMS REC REFERENCE NUMBER: Jan17/B/111.
